# Would loss to follow-up bias the outcome evaluation of patients operated for degenerative disorders of the lumbar spine? A study of responding and non-responding cohort participants from a clinical spine surgery registry

**DOI:** 10.3109/17453674.2010.548024

**Published:** 2011-02-10

**Authors:** Tore K Solberg, Andreas Sørlie, Kristin Sjaavik, Øystein P Nygaard, Tor Ingebrigtsen

**Affiliations:** ^1^Department of Neurosurgery, University Hospital of Northern Norway, Tromsø; ^2^Norwegian Registry for Spine Surgery (NORspine), Northern Norway Regional Health Authority, Tromsø; ^3^National Centre for Spinal Disorders and Department of Neurosurgery, University Hospital of St. Olav, Trondheim; ^4^Institute of Clinical Medicine, University of Tromsø, Norway

## Abstract

**Background and purpose:**

Loss to follow-up may bias the outcome assessments of clinical registries. In this study, we wanted to determine whether outcomes were different in responding and non-responding patients who were included in a clinical spine surgery registry, at two years of follow-up. In addition, we wanted to identify risk factors for failure to respond.

**Methods:**

633 patients who were operated for degenerative disorders of the lumbar spine were followed for 2 years using a local clinical spine registry. Those who did not attend the clinic and those who did not answer a postal questionnaire—for whom 2 years of outcome data were missing—and who would be lost to follow-up according to the standard procedures of the registry protocols, were defined as non-respondents. They were traced and interviewed by telephone. Outcome measures were: improvement in health-related quality of life (EQ-5D), leg pain, and back pain; and also general state of health, employment status, and perceived benefits of the operation.

**Results:**

We found no statistically significant differences in outcome between respondents (78% of the patients) and non-respondents (22%). Receipt of postal questionnaires (not being summoned for a follow-up visit) was the strongest risk factor for failure to respond. Forgetfulness appeared to be an important cause. Older patients and those who had complications were more likely to respond.

**Interpretation:**

A loss to follow-up of 22% would not bias conclusions about overall treatment effects and, importantly, there were no indications of worse outcomes in non-respondents.

Clinical registries are increasingly being used to monitoring treatment effectiveness and for evaluation of risk factors associated with different outcomes. Loss to follow-up may seriously bias the outcome assessments of clinical registries, and will reduce the statistical power due to smaller sample size (Hunt and White 1998, Hollis and Campbell 1999, Parker and Dewey 2000, Shih 2002, Gluud 2006). Information about outcomes of patients who do not respond at follow-up is valuable both for clinicians and researchers. In limited clinical trials, one can make vigorous attempts to trace and retain cohort members. Such efforts would be too expensive and resource-demanding in large population-based registries (Roder et al. 2005, Fritzell et al. 2006). Thus, researchers who use registry data will have to deal with higher numbers of non-respondents being lost to follow-up (Hunt and White 1998). If the outcomes of non-respondents and respondents are different, wrong conclusions could be drawn about the beneficial and harmful effects of interventions (Gluud 2006). Several studies have indicated that individuals who drop out of clinical trials have worse outcomes than those who do not (Sims 1973, Murray et al. 1997, Norquist et al. 2000, Ludemann et al. 2003, Kim et al. 2004). Different imputation methods have been developed to compensate for missing outcomes (Rubin and Schenker 1991, Little and Yau 1996, Shih and Quan 1997, Wood et al. 2004), but these methods are also susceptible to bias, since they rely on assumptions made about the dropouts (Hollis and Campbell 1999, Shih 2002). Studies of the “true” outcomes in non-respondents may help us to make the right assumptions about outcomes of patients who are lost to follow-up. In addition, to prevent loss to follow-up, we need information about risk factors for failure to respond.

Here we present a prospective study of patients who were operated for degenerative disorders of the lumbar spine. We assessed the outcomes of non-respondents, who would be lost to follow-up according to the standard procedures of registry protocols, and compared their outcomes with those of patients who responded, in order to evaluate whether the missing outcomes would bias conclusions about treatment effectiveness. We also wanted to identify risk factors for failure to respond.

## Patients and methods

### Study population

This study comprised all consecutive patients (n = 633) registered with 1 operation for degenerative disorders of the lumbar spine at the Department of Neurosurgery, University Hospital of Northern Norway (UNN), from Jan 1, 2000 through Dec 31, 2003 (Figure). Data collection and registration was part of the daily routines of the department, involving the entire staff, and the study population represented the total population operated and included in the registry at the unit (Solberg et al. 2005a, b).

**Figure.**
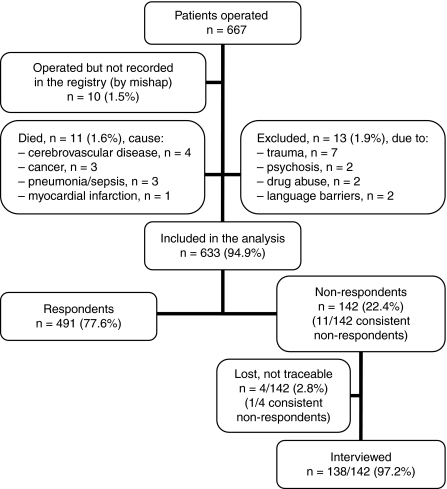
Study population.

The mean age of the patients (63% men) was 45 (16–83) years (Table 1). All patients were operated at 1 or 2 levels between L2 and S1. 557 (88%) were operated for the first time, and 76 (12%) had been operated previously. Of these 76 patients, 47 (62%) were reoperated at the same level, 25 (33%) at different level(s), and 4 (5%) were reoperated at both the same and different level(s). Follow-up time from the date of operation (baseline) was 2 years. The registry database was linked to the National Population Registry of Norway through the national 11-digit personal identification number. In this way, we obtained continuously updated information about changes of home address and dates of death in the study population. Causes of death were available from the medical records of the hospitals in our region.

**Table 1.** Characteristics of the study population

	All n= 633	I Respondents n =491	II Non-respondents n= 142	III **^a^** Consistent non-respondents n = 12	p-value of difference	
					I vs. II	I vs. III
Age, median (95% CI)	42 (41–44)	43 (41–44)	40 (37–44)	34 (29–51)	0.04	0.04
Females (%)	233 (36.8)	187 (38.1)	46 (32.4)	4(33.3)	0.2	0.7
BMI, median (95% CI) kg/m^2^^**b**^	26 (25–26)	26 (25–26)	26 (25–26)	25 (22–27)	0.7	0.5
Smokers (%)	296 (47)	224 (46)	71 (50)	8 (67)	0.4	0.2
Living alone, n (%)	171 (27)	126 (26)	45 (32)	8 (67)	0.2	0.002
Had child less than 8 years, n (%)	178 (28)	133 (27)	45 (32)	3 (25)	0.3	0.9
Weeks on sick leave, median (95% CI)	8 (5–11)	8 (6–12)	4 (1–13)	1 (0–24)	0.1	0.2
Days of hospital stay, median (95%CI)	4 (4–4)	4 (4–4)	3 (3–4)	3 (2–4)	0.02	0.1
Previous low back operation, n (%)	76.0 (12)	64 (13)	12.0 (9)	1 (8)	0.1	0.6
Had complication to the surgery, n (%)	31 (5)	29 (6)	2 (1)	0	0.03	0.4
EQ-5D score, median (95% CI)	0.16 (0.12–0.19)	0.16 (0.09–0.19)	0.16 (0.09–0.33)	0.23 (-0.10–0.69)	0.4	0.9
Health state, median (95% CI)	40 (35–40)	40 (35–40)	43 (38–50)	36 (22–55)	0.08	0.9
Leg pain, median (95% CI)	67 (64–69)	68 (65–70)	65 (60–69)	59 (26–84)	0.5	0.6
Back pain, median (95% CI)	54 (50–60)	55 (50–61)	52 (45–57)	51 (45–83)	0.1	0.6
Were anxious and/or depressed, n (%) **^c^**	304 (48)	231 (47)	73 (51)	8 (67)	0.4	0.2
Educational level, n (%)					1.0 **^d^**	0.8 **^d^**
Primary school	189 (30)	148 (30)	41 (29)	5 (42)		
Vocational school	218 (34)	168 (34)	50 (35)	3 (25)		
Gymnasium / high school	74 (12)	60 (12)	14 (10)	1 (8)		
University or college < 4 years	81 (13)	66 (13)	15 (11)	1 (8)		
University or college > 4 years	71 (11)	49 (10)	22 (16)	2 (17)		
Employment status, n (%)					0.7 **^e^**	0.8 **^e^**
On sick leave	345 (55)	276 (56)	69 (49)	6 (50)		
On partial sick leave	33 (5)	24 (5)	9 (6)			
Working full time	79 (13)	54 (11)	25 (18)	2 (17)		
Homemaker	10 (2)	9 (2)	1 (1)			
Student	28 (4)	12 (4)	7 (5)	1 (8)		
Unemployed	9 (1)	7 (1)	2 (1)	1 (8)		
Retired pensioner	62 (10)	53 (11)	9 (6)			
On rehabilitation ^**f**^	27 (4)	19 (4)	8 (6)			
Disability pensioner	40 (6)	28 (6)	12 (9)	2 (17)		
ASA grade I–V, n (%) **^g^**					0.8 **^h^**	0.4 **^h^**
Grade I	135 (40)	116 (36)	48 (40)	2 (22)		
Grade II	189 (57)	192 (59)	69 (57)	7 (78)		
Grade III	10 (3)	17 (5)	4 (3)			

**^a^** Group III was a subgroup of group II.

**^b^** Body mass index.

**^c^** Mild to severe problems.

**^d^** University or college education? (yes/no).

**^e^** On full or partial sick leave, on rehabilitation, or disability pensioner? (yes/no).

**^f^** Patients having received worker's compensation for more than 12 months with the prospect of returning back to work, or permanent disability status.

**^g^** No patients had ASA grade > III.

**^h^** ASA grade I vs. grade II and III.

We excluded participants who died within 2 years of follow-up. The causes of death were not related to the initial surgery. However, 1 patient (aged 67) died 26 days after the operation, of an acute myocardial infraction. We excluded 13 patients whose outcome evaluations would be biased by other severe, conflicting problems—as described in Figure.

Informed consent was obtained from all participants. The registry protocol was approved by the Data Inspectorate of Norway.

### Registry protocols/follow-up

In the year 2000, a comprehensive clinical spine surgery registry for quality control and research was established at UNN. Based on experiences from the Swedish Spinal Register (SweSpine) (Fritzell et al. 2006) and previous validation studies from the local clinical registry at UNN (Solberg et al. 2005a, Solberg et al. 2005b), the local registry of UNN was expanded to a national registry in 2007: the Norwegian Registry for Spine Surgery (NORspine). We have evaluated data obtained from the 2 protocols of the local registry at UNN. Protocol A was used in 2000 and 2001 and was changed to protocol B, which was used in 2002 and 2003. The only difference between the two protocols was how data were collected at 2 years of follow-up. Patients operated before 2002 (protocol A) were summoned for follow-up visits at the outpatient clinic at 24 months, whereas patients operated later (protocol B) received postal questionnaires. We could therefore investigate how these differences in obtaining follow-up data influenced response rates.

All patients were summoned for follow-up visits at 3 and 12 months at an outpatient clinic. The questionnaires and a stamped, addressed return envelope were distributed by ordinary postal mail, to be completed at home by the patients. An independent observer, a research nurse responsible for all follow-up visits, collected and checked all the returned questionnaires and interviewed the patients about employment status and complications. Travel expenses were covered by the public National Insurance Organization.

At 2 years, patients who did not attend the clinic (protocol A) got one reminder by telephone within a few days, from the research nurse. They were asked to make a new appointment for a follow-up visit or to respond by postal mail. Patients who did not return the questionnaire at 2 years (protocol B) got 1 reminder with a new copy of the postal questionnaire and a stamped, addressed return envelope.

### Respondents/non-respondents

Patients for whom 2-years of follow-up data were missing, despite these measures, would be lost to follow-up under standard protocol conditions. They were defined as non-respondents (group II, n = 142; protocol A, n = 37; protocol B, n = 105) and they were invited to participate in the study by telephone interview. Patients who did not respond at 3, 12, or 24 months were classified as consistent non-respondents (group III, n = 12: protocol A, n = 8; protocol B, n = 4). Thus, group III was a subgroup of group II. The rest of the patients were defined as respondents (group I, n = 491) (Figure).

We used 3 sources for tracing the non-respondents: the National Population Registry of Norway, publicly available online telephone directories (Harvey et al. 2003), and the electronic medical records of the hospital. 138 of the 142 non-respondents were interviewed by telephone in a standardized fashion (Hunt and White 1998) by the same interviewer (AS). These patients were instructed to report their condition at 2 years after surgery.

The patients were also asked to give their main reason for not responding. When data collection was complete, the study group had a consensus meeting where patients' answers were categorized into 5 main reasons for not responding: “forgot to complete or return the questionnaire”, “questionnaire fatigue”, “sickness”, “could not remember having received questionnaires”, and “family- or work-related problems”.

### Baseline data

At admission, the patients completed the baseline questionnaire. During their hospital stay, the surgeon recorded data concerning diagnosis, treatment, employment status, and duration of symptoms according to a standard registration form. Finally, all questionnaires and forms were collected and checked for completeness by a dedicated research nurse.

### Questionnaires

The questionnaires completed by the patients at baseline and follow-up were identical, and were used for outcome assessments, including interviews. The baseline questionnaire contained additional questions about demographics and lifestyle issues. The primary outcome measure was the EuroQol-5D (EQ-5D) questionnaire. Secondary outcome measures were perceived benefit of the operation, employment status, and visual analog scales (VAS) for leg pain, back pain, and state of health.

### EQ-5D

EQ-5D is a generic and preference-weighted measure of health-related quality of life (HRQL). It evaluates 5 dimensions: mobility, self-care, activities of daily life, pain, and anxiety and/or depression. For each dimension, the patient describes 3 possible levels of problems (none, mild to moderate, or severe). Hence, this descriptive system contains 243 (3^5^) combinations or index values for health states (the EuroQol Group 1990). We used the value set based on the main survey from the EuroQol group (Dolan et al. 1996, Dolan 1997), which has been validated for this patient population (Solberg et al. 2005b). Total range of score is from –0.594 to 1, where 1 corresponds to perfect health and 0 to death. Negative values are considered to be worse than death (the EuroQol Group 1990).

### Health state

EuroQol VAS forms the second part of the EQ-5D questionnaire. The patients rate their general state of health by drawing a line from a box marked “your health state today” to the appropriate point on the 20-cm VAS scale, which ranges from 0 to 100 (worst to best imaginable health) (the EuroQol Group 1990).

### Benefit of the operation

At follow-up, the patients were asked: “How much benefit have you had from the operation?” The response alternatives were “very much”, “quite a lot”, “some”, “none at all” or “uncertain” (Solberg et al. 2005a, b).

### Leg pain and back pain

Pain intensity was graded by the patient in 2 separate 100-mm VAS for leg and back pain (where 0 = no pain).

### The American Society of Anesthesiologists (ASA) grading system

ASA grade was registered for each patient by a doctor or a specialized nurse before surgery. ASA grade (I–V) classifies patients according to their vulnerability, i.e. physical condition (from no disease to life-threatening systemic disease) (Dripps 1963). Before 2002, data on ASA grade were not registered systematically (62% missing data), and they were therefore omitted from the analysis. Of the data from 2002 and 2003, only 9% were missing. These values (except 1) could be obtained from the medical records of the patients.

### Statistics

We tested whether within-group change scores were statistically significant (change from baseline to follow-up), using paired t-test or Wilcoxon's matched-pairs signed rank test depending on the distribution of the data. Baseline characteristics and differences in outcome between subgroups (I–III) were assessed with independent-samples t-test, Mann-Whitney U-test, or Chi-square test. Central tendency is presented as mean when normally distributed, and as median when skewed. Confidence intervals for medians were calculated according to McKean and Schrader (1984). We assessed risk factors for not responding at 2 years of follow-up in multivariate analysis, using respondents (value = 0) vs. non-respondents (value = 1) as dependent variable. Being summoned for a follow-up visit (protocol A) vs. receiving a postal questionnaire (protocol B) was used as exposition variable. We adjusted for covariates obtained from baseline data (Table 1) using a backward logistic regression model, only if the covariates were judged to be clinically relevant and if baseline values differed significantly (level 0.1) between respondents and non-respondents.

To get a better model-data fit, we had dichotomized two covariates: living alone and complications (yes/no). SPSS for Windows version 14.0 was used for all analyses.

## Results

Non-respondents were younger, were hospitalized for fewer days, and had more complications than the respondents. Consistent non-respondents were more likely to live alone (Table 1). We found no difference in ASA grade between the groups. However, this result is uncertain since we lacked data from 2000 and 2001, when the response rate was highest. Disc herniation treated by microdiscectomy was the commonest operation (Table 2).

**Table 2.** Indications for and types of surgery among respondents non-respondents

Group	All n = 633	I Respondents n = 491	II Non-respondents n = 130	III **^a^** Consistent non-respondents n = 12
Indications for surgery, n (%)				
Lumbar disc herniation	519 (82)	399 (81)	120 (84)	12 (100)
Central spinal stenosis	94 (15)	78 (16)	16 (11)	
Lateral spinal stenosis	39 (6)	31 (6)	8 (6)	
Segmental instability	31 (5)	24 (5)	7 (5)	
Sum **^b^**	683	532	151	12
Types of surgery, n (%)				
Microdiscectomy	476 (75)	362 (74)	114 (80)	11 (92)
Laminectomy	111 (17)	90 (18)	21 (15)	
Instrumented fusion	30 (5)	24 (5)	6 (4)	
Chemonucleolysis	16 (3)	15 (3)	1 (1)	1 (8)
Sum	633	491	142	12

**^a^** Group III was a subgroup of group II.

**^b^** Patients could have more than one indication for surgery.

### Response rates

The overall response rate declined during the follow-up period, to 77.6% at 24 months. When the protocol was changed from A to B in 2002, the response rate decreased considerably. Patients who were invited for a follow-up visit at the outpatient clinic at 2 years (protocol A) had a higher response rate than patients who only received questionnaires by mail (protocol B) (88% vs. 69%, p < 0.001).

4 patients could not be traced (Figure); among them, 1 was a consistent non-respondent. After obtaining the missing outcomes of the non-respondents by telephone interview, the outcome data were 99% complete (Table 3). None of the non-respondents refused to be interviewed.

**Table 3.** Sequential outcomes of the study population during 2 years of follow-up

Follow-up	3 months		12 months		2 years	
n (response rate)	598 (95%)		574 (91%)		629 (99%) **^a^**	
EQ-5D score **^b^**	0.45 (0.41–0.48)	< 0.001	0.46 (0.43–0.50)	< 0.001	0.46 (0.43–0.49)	< 0.001
Health state **^b^**	29 (27–31)	< 0.001	31 (29–34)	< 0.001	30 (28–32)	< 0.001
Leg pain **^b^**	43 (41–46)	< 0.001	41 (38–44)	< 0.001	41 (38–44)	< 0.001
Back pain **^b^**	29 (27–31)	< 0.001	28 (25–31)	< 0.001	27 (24–30)	< 0.001
Benefited from the operation, n (%) **^c^**	539 (90)		527 (92)		571 (91)	
Received worker's compensation, n (%) **^d^**	335 (57)		166 (31)		181 (29)	

**^a^** Includes non-respondents interviewed by telephone.

**^b^** Absolute values (improvements from baseline) are shown as mean change, (95% CI) and p-value

**^c^** Patients who stated that they had “some”, “much”, or “very much” benefit from the operation.

**^d^** Patients who were on full or partial sick leave, on rehabilitation, or disability pensioners.

To trace and interview non-respondents was time consuming. The mean time from the operation until all the data concerning 24 months of follow-up had been collected was 2 years for the respondents and 3 years for the non-respondents.

We identified 5 main reasons for not responding: forgot to complete or return the questionnaire (n = 87, 63%), questionnaire fatigue (n = 23, 17%), sickness (n = 15, 11%), could not remember having received questionnaires that had been sent (n = 7, 5%), and family- or work-related problems (n = 5, 4%). Information from 1 patient was missing.

### Outcome assessment

Both primary and secondary outcome measures improved after the operation. These effects persisted throughout the observation period (Table 3).

There were no statistically significant differences in outcome between respondents and non-respondents or between respondents and consistent non-respondents, measured by employment status and perceived benefits of the operation at 2 years of follow-up, and improvements in HRQL, health state, leg pain, and back pain (Table 4).

**Table 4.** Subgroup analyses of respondents and non-respondents at 2 year

Outcome **^a^**	I. Respondents n = 491 (77.6%)		II. Non-respondents n = 138 (21.8%)		III. Consistent non-respondents **^b^** n = 11		p-value of difference	
							I vs. II	I vs. III
EQ-5D score **^c^**	0.46 (0.36–0.60)	< 0.001	0.41 (0.30–0.64)	< 0.001	0.64 (0.19–0.76)	0.003	0.8	0.6
Health state **^d^**	31 (28–34)	< 0.001	27 (22–32)	< 0.001	28 (13–43)	0.002	0.1	0.7
Leg pain **^d^**	40 (37–43)	< 0.001	44 (38–50)	< 0.001	43 (25–61)	0.001	0.3	0.8
Back pain **^c^**	22 (18–28)	< 0.001	26 (17–32)	< 0.001	40 (17–66)	0.008	1.0	0.3
Benefited from the operation, n (%) **^e^**	447 (91)		124 (91)		11 (100)		0.8	0.3
Received workers compensation, n (%) **^f^**	141 (29)4		0 (29)		4 (36)		1.0	0.6

**^a^** Improvements from baseline (absolute values) are shown.

**^b^** Group III is a subgroup of group II.

**^c^** Median change, (95% CI) and p-value

**^d^** Mean change, (95% CI) and p-value

**^e^** Patients who stated that they had “some”, “much”, or “very much” benefit from the operation.

**^f^** Patients who were on full or partial sick leave, on rehabilitation, or disability pensioners.

For the non-respondents, there were no statistically significant differences in outcomes between those who did not attend the outpatient clinic (protocol A) and those who did not respond to a postal questionnaire (protocol B) (data not shown).

### Complications

31 patients (5%) had 34 complications (Table 5). Complications were more frequent among the respondents than among the non-respondents (7% vs. 1%, p = 0.03).

**Table 5.** Types of complications in 31 (5%) of 633 patients ^a^

Complications	All n = 633	I. Respondents n = 491	II. Non-respondents n = 142
Dural tear	9	9	
Deep wound infection	5	5	
Superficial wound infection	10	9	1
Urinary bladder infection	2	2	
Reoperation within the same hospital stay	2	2	
Intraoperative nerve root injury	1	1	
Postoperative muscle hernia	1		1
Gall bladder infection	1	1	
Deep leg vein thrombosis	1	1	
Gastric ulcer hemorrhage	1	1	
Minor myocardial infarction	1	1	
Sum, n (%)	34 (6)	32 (7)	2 (1)

**^a^** 3 of the patients had 2 complications. No complications occurred in consistent non-respondents (group III).

### Risk factor analysis

2 independent risk factors for failure to respond were found by multivariate analysis (Table 6). Patients (operated in 2002 and 2003) who only received postal questionnaires (protocol B) at 2 years of follow-up were less likely to respond than those who were summoned for a follow-up visit at the outpatient clinic (protocol A) (odds ratio (OR) = 3, 95% CI: 2–5). A 1-year increase in age increased the probability of responding by 2% (OR = 0.98). Having had a complication and living alone were not independent risk factors in the multivariate analysis (Table 6).

**Table 6.** Risk factors for failure to respond at 2 years of follow-up in 633 patients

Factors	OR **^a, b^**	95% CI	p-value	OR **^c^**	95% CI	p-value
Only received postal questionnaires at 2 years **^d^**	3.2	(2.1–4.9)	< 0.001	3.2	(2.1–4.9)	< 0.001
Age	0.99	(0.97–1.0)	0.05	0.98	(0.97–1.0)	0.05
Had any complication	0.23	(0.54–1.0)	0.05	0.26	(0.06–1.1)	0.07
Days of hospital stay	0.89	(0.82–0.96)	0.01	0.99	(0.90–1.1)	0.9
Health state	1.0	(0.99–1.0)	0.1			
Living alone	1.3	(0.89–2.0)	0.2			

**^a^** OR: odds ratio.

**^b^** Univariate analysis.

**^c^** Multivariate analysis.

**^d^** Protocol A, operated in 2000 and 2001.

## Discussion

We found similar outcomes between respondents and non-respondents at 2 years of follow-up in patients who were operated for degenerative disorders of the lumbar spine, assessed as changes in HRQL (EQ-5D) score, pain, and state of health, or employment status and perceived benefit. Importantly, the non-respondents did not have poorer outcomes than the respondents. However, better outcome in consistent non-respondents might have reached statistical significance if the sample size had been larger. The patients reported forgetfulness as the main reason for not responding. The patients most likely to respond were those who were summoned for follow-up visits and older patients.

It has been suggested that as a rule of thumb, a loss to follow-up of greater than 20% probably leads to assessment bias, whereas a rate of less than 5% would not (Sackett et al. 2000, Schulz and Grimes 2002). Our results indicate that a 22% loss to follow-up does not alter the conclusions about the overall effects of treatment within the whole, large cohort. In statistical terms, we could treat the non-respondents as if they were missing at random (Shih 2002). However, by simply ignoring the non-respondents, somewhat older patients and those who had complications would be over-represented. Where there were lower response rates, this could confound the overall assessments towards poorer treatment effects if older patients and those who had complications tended to report poorer outcomes. To prevent selection bias, for example when comparing subgroups of patients with different response rates, the treatment effects should be adjusted for clinically relevant risk factors associated with responding (Etter and Perneger 1997, Wood et al. 2004).

The safest way to avoid bias is to reduce loss to follow-up. Our study shows that patients who only received postal questionnaires were 3 times less likely to respond than those who were summoned for follow-up visits. Similar results have been published previously (Sitzia and Wood 1998). It would be too demanding on resources to arrange long-term follow-up visits for the participants in large clinical registries (Roder et al. 2005, Fritzell et al. 2006). The patients would therefore have to be contacted at home. Several ways of increasing response rates to postal questionnaires have been recommended (Etter and Perneger 1997, Edwards et al. 2002, 2007, Etter et al. 2002, Schulz and Grimes 2002). We found that forgetfulness was the most important reason for failure to respond. This problem can be prevented by sending early reminders to study participants, for example by using modern telecommunication. SMS and e-mail are now widely available, especially to younger patients who are less likely to respond.We assessed a homogenous patient population living in a typical Northern European society where most public health services are free, national population registries are updated, and the level of social security is high. Thus, people from lower socioeconomic classes and patients with disability can afford to respond, and can be given help to respond. This might explain why we did not find worse outcomes in the non-respondents. Our findings may not be valid for populations living under other ethnic and socioeconomic conditions.

One weakness of this study is that only non-respondents were interviewed by telephone, with a time delay of 12 months. The delayed interviews may have introduced recall bias. However, previous reports on sequential long-term outcomes in similar patient populations have shown that the outcomes are relatively stable (Findlay et al. 1998, Amundsen et al. 2000, Atlas et al. 2000). Thus, we would expect recall bias to be small. Some studies have indicated that interview subjects tend to overestimate favorable outcomes (Burroughs et al. 2001, Ludemann et al. 2003), but the opposite has also been suggested (Wildner 1995). In our study, the non-respondents did not report better outcomes, even though they were somewhat younger and had fewer complications than patients who responded. It was beyond the scope of this study to evaluate assessment bias due to deaths in study participants. Cohort members who die during follow-up must be accounted for and handled separately in the analyses, as previously described (Lachin 1999, Shih 2002).
